# Inferential accuracy in classroom contexts

**DOI:** 10.3389/fpsyg.2025.1647219

**Published:** 2025-11-07

**Authors:** Zachary J. Schroeder, Jenefer Husman, Sara D. Hodges

**Affiliations:** 1 Department of Psychology, College of Arts & Sciences, University of Oregon, Eugene, OR, United States; 2 Department of Education Studies, College of Education, University of Oregon, Eugene, OR, United States

**Keywords:** inferential accuracy, empathic accuracy, pedagogical content knowledge, informal formative assessment, discretionary spaces

## Abstract

This paper connects two research traditions—social psychology's examination of inferential accuracy, and educational research on teacher cognition and decision-making, in order to consider how teachers attempt to accurately infer their students' thoughts and feelings during instruction. Our aim is to link the social psychological study of inferential accuracy with several prominent educational constructs related to teacher expertise. We begin by introducing inferential accuracy and providing a brief overview of social psychology research about it. We suggest that inferential accuracy may affect teacher decisions in what are known as “discretionary spaces” in classroom settings. Next, we delineate similarities and differences between inferential accuracy and two constructs related to teacher expertise (diagnostic competence and teacher noticing). We then identify the parallels between inferential accuracy and two other educational constructs—pedagogical content knowledge and informal formative assessment. Throughout this synthesis, we consider applications of inferential accuracy research to inform how, and how well, teachers may be able to infer their students' thoughts and feelings during instruction, as well as the ways that social psychology researchers may consider teacher-student classroom interactions as fruitful settings in which to examine interpersonal accuracy. We point out the educational constructs that correspond to social psychological predictors of inferential accuracy, discuss areas of tension when translating inferential accuracy research into educational contexts, and suggest future research questions that may prove fruitful with interdisciplinary collaboration.

## Introduction

How do teachers know the right thing to do next in the classroom? The methods of defining, operationalizing, and measuring teacher expertise are as varied as the tasks teachers undertake before, during, and after instruction (Anderson and Taner, 2023; Berliner, 2001; Bromme, 2001; Clarke et al., 2011; Dunham, 2002; Eraut, 2007; Gotwals et al., 2015; Hattie, 2009; Palmer et al., 2005; Ropo, 2004; Shulman, 2000; Tsui, 2005, 2009; van Dijk et al., 2020). One particularly difficult-to-study aspect of teacher expertise is how teachers make moment-to-moment decisions not structured within a predetermined lesson plan—decisions that are in part informed by teachers' inferences about what is going on in the heads of their students (e.g., choosing which student to call on, deciding how to address misbehavior, or determining whether to move forward with a lesson in lieu of taking extra time to review a concept). Navigating these rapid-fire decisions, called *discretionary spaces* (Ball, 2018), requires teachers to recruit a diverse set of pedagogical, socio-emotional, and interpersonal skills and knowledge (e.g., pedagogical content knowledge, individuated knowledge about their students, meta-awareness of how they are perceived by the class).

In this conceptual analysis, we review social psychological research on *inferential accuracy* (the accurate inference of a target's thoughts and/or feelings, Hodges et al., 2024, 2015; Ickes et al., 1990), which has (to our knowledge) never been examined in the context of teachers during instruction. Inferential accuracy may offer a unique perspective for education researchers to examine how discretionary space choices and other classroom decisions are informed by teachers' inferences about students' dynamic thoughts and feelings. We first introduce inferential accuracy with a brief background and then recount an example of two discretionary spaces from an elementary school math class to provide an illustration and context for our discussions of educational and psychological constructs. We discuss *grain-size* as a means of contrasting inferential accuracy with two similar constructs from education research, diagnostic competence and teacher noticing (Ball, 2018; Wilson, 2024). We next examine the overlap and tension between inferential accuracy and two educational constructs related to teacher expertise: pedagogical content knowledge and informal formative assessment. In addition, we identify educational constructs that correspond to social psychological predictors of inferential accuracy to orient the reader to inferential accuracy's possible place in classroom research. We also discuss areas of tension when translating inferential accuracy research into educational contexts and suggest future research questions that may prove fruitful with interdisciplinary collaboration. Throughout the paper, we seek to build a bridge between one form of interpersonal accuracy studied by social psychology (inferential accuracy) and several prominent educational constructs reflective of teacher expertise in pedagogical contexts. These educational constructs are themselves wide-reaching and heterogeneous and so we limit the main focus of this paper to a conceptual review of their relation to inferential accuracy.

### Inferential accuracy

Inferential accuracy is the accurate inference by one person (the “perceiver”) of another person's (the “target's”) thoughts and feelings during a social interaction (Hodges et al., 2015; Ickes, 2016). It has historically been referred to as “empathic accuracy,” although recent calls (e.g., Hodges et al., 2024) have suggested moving away from this term because it incorrectly suggests that this accuracy always leads to prosocial outcomes (Consider, as an example, a poker player who uses inferential accuracy to aid in taking an opponent's money). Within the focus of the current manuscript, we view teachers as perceivers (i.e., the ones inferring someone else's thoughts) and students as targets (i.e., the ones whose thoughts are inferred). Although inferences likely occur in both directions (i.e., students may also attempt to infer the thoughts and feelings of their teachers; see Gleason et al., 2009), the current paper is specifically interested in teacher inferences of students' thoughts and feelings.

#### The Ickes paradigm

The most common paradigm for studying inferential accuracy was developed by William Ickes and colleagues and first appeared in publications in the 1990s (Ickes et al., 1990). In this paradigm, two participants—i.e., a “dyad,” (defined as a group consisting of two people, particularly two people in a social interaction^1^)—engage in a video-recorded verbal interaction. Immediately following the interaction, one participant (the target) watches the video, which is paused intermittently at which times the targets report what they were thinking and feeling. The intermittent pauses may occur when the target reports remembering discrete thoughts/feelings or at predetermined intervals (e.g., every 30 s). The other participant (the perceiver) then watches the video of the interaction and, at the times when the target reported their thoughts and feelings, the perceiver is asked to infer—and provide in writing—their best guess of what the target was thinking at that moment (Gesn and Ickes, 1999; Ickes, 2016; Ickes et al., 1990). These thoughts and feelings—reported by the target and inferred by the perceiver—are then compared and rated for accuracy on a 3-point or 4-point scale by a team of coders.

The Ickes paradigm has developed into two sub-paradigms, both of which are relevant to the points we make in this paper, as both share the fundamental element of a perceiver inferring the thoughts and feelings of a target person, which we believe is the key ingredient that makes inferential accuracy interesting to contemplate in classrooms. In the *dyadic interaction paradigm*, the perceiver infers the thoughts and feelings of a target with whom they just interacted. In the *standard stimulus paradigm*, the perceivers can infer the thoughts and feelings of a target with whom they do not directly interact; instead, the target is viewed in a video recording interacting with another conversation partner (e.g., see Marangoni et al., 1995). In the standard stimulus paradigm, targets still re-watch their own videos to report their thoughts and feelings, but perceivers can be recruited to watch those videos and to infer the targets' thoughts and feelings at research sessions in the future, at times and in settings far removed from those at which the interaction in the video initially took place. Researchers use these different paradigms for different purposes (e.g., the standard stimulus paradigm aids in identifying target-level effects by having multiple perceivers view a single target; it also provides a standard test of accuracy, allowing comparison of different categories of perceivers or perceivers taking part under different conditions. The dyadic interaction paradigm allows for the examination of variables related to the target and perceiver's unique relationship—e.g., its length and quality.

#### Predictors of inferential accuracy

Although target characteristics can contribute to accuracy (Human and Biesanz, 2013; Lewis, 2014), the predominant focus of past inferential accuracy research has been identifying predictors of accuracy associated with the perceiver (i.e., what makes a good “mind-reader”). From this work, three predictors of perceiver accuracy have garnered robust empirical support: (1) the perceiver's familiarity with the target; (2) the use of cognitive schemas when making inferences; and (3) the use of verbal information from the target. Although inferential accuracy between teachers and students has not been studied in the context of education, each of these sources of accuracy correspond to a construct within teacher expertise, suggesting that expert teachers should be more accurate in inferring their students' thoughts in discretionary spaces.

First, familiarity with the target improves accuracy: friends are better at inferring a target's thoughts and feelings than strangers (Stinson and Ickes, 1992) and inferential accuracy generally increases as perceivers see more of a target (Marangoni et al., 1995). Curiously, in zero-acquaintance education research (studies conducted where teachers are unfamiliar with the students, e.g., Bhowmik et al., 2025), teachers' accuracy for students' stable characteristics after seeing a 30-s video is comparable to their accuracy in a natural classroom, suggesting that their expertise allows for accurate, rapid-fire judgments of stable characteristics or student characteristic profiles (Nickerson, 1999; Seidel et al., 2021). As described above, however, the relationship between a teacher's familiarity with their students and their accuracy for variable emotions or specific thought content at the level at which they are studied in inferential accuracy (e.g., every 30 s) remains understudied (Borko et al., 2008).

Second, incorporating schemas about people (and more specifically schemas that are actually validly informative) into inferences about a specific target's thoughts and feelings is related to increased accuracy (Hodges and Kezer, 2021; Lewis et al., 2012; Stinson and Ickes, 1992). “Informative schemas” are schemas about people that are made up of accurate information about a particular individual (Stinson and Ickes, 1992) or about categories of people (Lewis et al., 2012)^2^. As we discuss in detail later, schemas in educational contexts associated with increased accuracy may include a teacher's generalizations about how students at a particular developmental stage or who come from a certain kind of educational background may encounter common pitfalls when learning a specific topic—in other words, drawing on teachers' pedagogical content knowledge.

Third, the utilization of verbal information from the target (i.e., what the target says) corresponds to higher inferential accuracy. Multiple studies have shown that access to the target's words leads to higher accuracy—even in the absence of the video of the target (Gesn and Ickes, 1999; Hall and Schmid Mast, 2007; Hodges and Kezer, 2021; Kraus, 2017). Generally, attending to and using what targets say improves inferential accuracy in part because target speech tends to correspond to what targets are thinking (Hodges and Kezer, 2021). In the classroom, explicitly *asking* targets (students) to verbalize what they are thinking is a well-supported pedagogical tool that is a part of informal formative assessment, which we discuss below. Armed with this basic overview of how inferential accuracy has been studied, and importantly, sources of information that perceivers may use to accurately infer a target's thoughts, we now consider the educational concept of “discretionary spaces” and the decisions that teachers make in these spaces, to illustrate how inferential accuracy is likely to be reflected in student-teacher interactions within the classroom.

### Discretionary spaces

**Figure 1:**
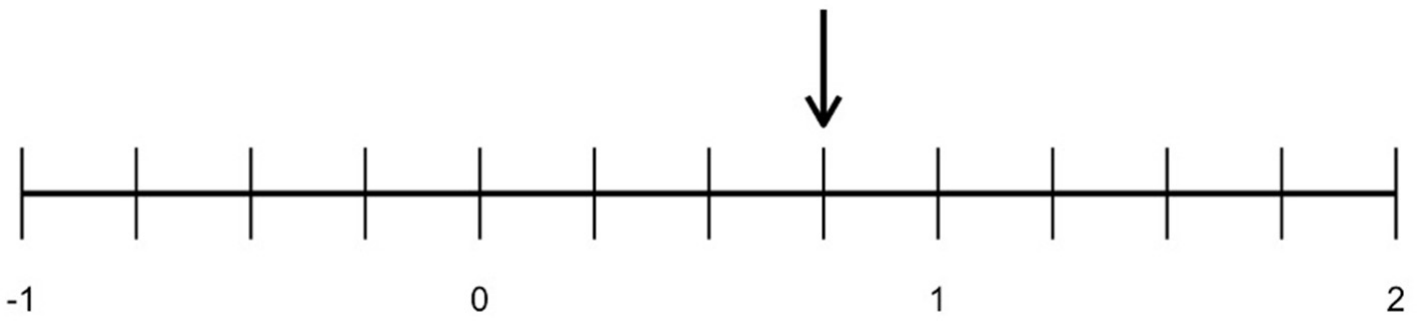
Fraction and number line task adapted from Ball (2018).

To illustrate the concept of discretionary spaces, we offer the case study Deborah Ball (2018) presented in her presidential address to the American Educational Research Association. In this example, an expert teacher (Ball herself) leads a math lesson about locating fractions on a number line in an American elementary school classroom. Ball begins by noting that this lesson is often difficult for students due to the inconsistency between how number lines are presented (as consisting of whole numbers) and how fractions are visualized (as parts or “slices” of pizzas and pies). The students in Ball's example are tasked with identifying the fraction, 3/4, which is pointed out by an arrow on a number line (Figure 1). One student is called on and reports that she labeled the fraction 7/8. Although incorrect, her answer demonstrates a common pitfall for students in the lesson: counting all dashes on the number line instead of anchoring at zero. This first student's explanation is interrupted by a second student who asks, “why'd you choose seven-eighths?”

Ball identifies 22 unique discretionary spaces in an 88-s portion of this math lesson. Although Ball does not specifically describe inferring either student's thoughts or feelings as part of her proximal deliberation when navigating these discretionary spaces, we saw two examples of how Ball's expertise informed her inferences of students' thoughts, which in turn influenced Ball's decisions: (1) Ball infers the first student's reasoning as illustrative of a common misconception and thus worth exploring with the class, and (2) Ball infers that the second student's interruption was caused by mathematical curiosity (not an attempt to taunt the first student for answering incorrectly) and so does not reprimand her. Although both decisions reflect Ball's pedagogical content knowledge and are made in service of overarching pedagogical goals, they are also made in a distinctly social context and require Ball to leverage socio-emotional and interpersonal skills and knowledge.

In the first discretionary space, Ball infers a student's thinking as exemplary of a common misconception, an accurate inference informed by her pedagogical content knowledge (discussed below). An inaccurate inference in that moment (perhaps made by a novice teacher) may have missed the cues to this student's thinking and overlooked an opportunity for collaborative learning. This discretionary space is complicated by the socio-emotional risks Ball takes in asking the student to share her erroneous thinking with the class. Accurately inferring the student's current thoughts and feelings may support a positive pedagogical opportunity in which the student shares willingly or is appropriately pushed beyond her comfort zone to speak in front of the class. However, given the many social, emotional, and academic pressures students feel when participating in math classes (e.g., Ashcraft, 2002; Good et al., 2012), inaccuracy could result in Ball requesting a student who may be frustrated with the lesson or insecure in their feelings of belonging to share a wrong answer publicly and feel humiliated or picked on, jeopardizing Ball's positive teacher-student relationship with that student (e.g., Klassen et al., 2012).

The second discretionary space requires Ball to leverage very different information to determine whether to reprimand the interrupting student or encourage a dialogue between her and the first student. This decision likely incorporates Ball's knowledge of both students' individual thinking, knowledge of the students' relationships to one another and to the other students in the class, and knowledge of classroom protocols (e.g., the second student raised her hand prior to speaking but did not wait to be called on). Ball opts not to reprimand this second student, accurately inferring that she was genuinely curious about the first student's thinking. Inappropriately reprimanding the student risks punishing her for expressing mathematical curiosity, but failing to intervene if the second student were taunting the first risks the first student's feelings. In both cases, improper action risks harming a student's feelings of belonging (Good et al., 2012; Lewis and Hodges, 2015).

These discretionary spaces require Ball to incorporate a wide variety of prior information and proximal cues when making pedagogical decisions. Yet, they are only a few seconds apart, occur at the same level of analysis (i.e., they both constitute one discretionary space), and both clearly reflect her expertise. In our examination of several lines of education research (discussed below), no prior studies have collected reports of what students were thinking and feeling in discretionary spaces such as these. This may be a missed opportunity, as by examining students' thoughts and feelings in discretionary spaces and comparing them to teacher inferences, researchers may access a novel operationalization of teacher expertise that can begin to demonstrate how teachers' personal and enacted pedagogical content knowledge impacts the accuracy of their inferences about students and which in turn influences the effectiveness of their judgments and actions in the classroom.

### Grain-size

To think about inferential accuracy in the classroom, we must consider the concept of grain-size. “Grain-size” is used in describing empirical research to differentiate levels and schedules of measurement—for example, to contrast large grain-size methods (e.g., teacher judgments as they are shaped over an entire academic year, Meissel et al., 2017) with small grain-size methods (e.g., measures of teachers' blood-oxygen level or heart-rate variability during classroom instruction that are taken every few seconds, Donker et al., 2023; Tobin et al., 2016; Wettstein et al., 2024). Grain-size encompasses not only the frequency of measurements but the scope and scale of the measures as well—it may include fine-tuned data such as heart-rate variability or complex, qualitative data from a teacher's reflections on a year of teaching.

The grain-size associated with discretionary spaces is particularly tricky for researchers as it encompasses moment-to-moment data for both teacher thinking *and* student experiences. In the case study example featured earlier in this paper, how researchers examine the second discretionary space (the student's interruption) may include recording data on Ball's proximal cognition of that student's rationale for interrupting her classmate and what the student is thinking in that moment (i.e., whether the student was taunting or positively engaging with her classmate).

At first glance, inferential accuracy in classroom contexts may seem to be already covered by two core constructs within teacher expertise: *diagnostic competence* (also referred to as diagnostic judgments or teacher judgment accuracy, Heitzmann et al., 2019; Helmke and Schrader, 1987; Loibl et al., 2020; Südkamp et al., 2012; Urhahne and Wijnia, 2021) and *teacher noticing* (also called professional vision, Jacobs et al., 2010; König et al., 2022; Sherin, 2014; Sherin et al., 2011). However, when considered in conjunction with grain-size, we believe that inferential accuracy taps into a precise—and understudied—empirical niche.

### Diagnostic competence

Diagnostic competence most often refers to a teacher's accurate judgment of comparatively stable characteristics, such as students' academic achievement. It is often assessed by comparing teacher judgments to standardized test scores (Demaray and Elliot, 1998; Helmke and Schrader, 1987; Südkamp et al., 2012), reading ability (Beswick et al., 2005) or student cognitive ability measures (Machts et al., 2016). Some work has examined diagnostic competence for more dynamic processes, such as learning behaviors (Klug et al., 2016) or accurate judgments of the appropriateness, difficulty, and applicability of a task for a specific learning outcome (for a review, see Urhahne and Wijnia, 2021). However, even though the characteristics that are often studied as part of diagnostic competence (e.g., reading ability, achievement) change over time, they can be considered relatively stable when compared to the moment-to-moment dynamic thoughts and feelings that are examined in inferential accuracy. Of the few studies of diagnostic competence that have examined more dynamic student states, the main focus has been on emotional experiences such as test anxiety or general feelings toward school (e.g., Zhu and Urhahne, 2021) and not on dynamic student mental states that change moment-to-moment within a lesson. Thus, inferential accuracy in the classroom could be viewed as a sub-construct of diagnostic competence that specifically examines teacher accuracy—one that occurs at a smaller grain-size than current measures employed to study diagnostic competence.

### Teacher noticing

The second construct within teacher expertise that bears similarity to inferential accuracy (but also differs from it) is teacher noticing—how a teacher makes sense of the “noise” of a classroom and attends to (or ignores) information relevant to their proximal pedagogical goals (Sherin, 2014; Sherin et al., 2011). An expert teacher viewing a classroom mid-lesson is like a chess expert viewing a chessboard mid-game: they notice important features of the environment, ignore unimportant features, and extract meaningful patterns that novices miss, (e.g., an expert teacher may ignore some student misbehavior that does not jeopardize the flow of the lesson just as an expert chess player may ignore pieces that they have already blocked—see Chase and Simon, 1973).

When measuring both inferential accuracy and teacher noticing, attention is paid to how a perceiver (i.e., teacher) processes cues from the environment, but, as with diagnostic competence, inferential accuracy involves a somewhat smaller grain-size than prevalent current measures of teacher noticing. The study of teacher noticing has most often addressed the level of the classroom and focuses on how teachers extract cues of student learning and misbehavior from the class as a whole (Berliner, 2001; König et al., 2022). Inferential accuracy, on the other hand, occurs at the level of discrete student thoughts. This level may be more familiar in dyadic pedagogical interactions such as one-on-one tutoring, but many discretionary spaces within a full-class may be viewed as dyadic—for example, consider Ball's decision in our example to call the first student forward to share her thinking. Although Ball may have considered the other students in the class, that decision was based largely on her specific inference about that one student's thinking. One sub-facet of teacher noticing research, informal formative assessment (e.g., Sezen-Barrie and Kelly, 2017), bears a striking concordance with research findings about inferential accuracy that support using what a target says as a basis for accurate thought-feeling inferences. As such, we discuss informal formative assessment in more detail below.

Thus, inferential accuracy encompasses a dyadic exchange between teacher and student (differentiating it from the bulk of teacher noticing research) and touches on a mixture of dynamic socio-emotional and pedagogical states—at a grain-size that is finer than the relatively-stable traits that make up the bulk of diagnostic competence research. This is not to say that these areas of research cannot inform one another, but instead to specify that inferential accuracy embodies diagnostic competence and/or teacher noticing specifically within the micro-moments that are discretionary spaces.

We turn next to two widely studied educational constructs—pedagogical content knowledge and informal formative assessment—that have been used to understand teacher expertise and that we also believe play a role in inferential accuracy. Both of these educational constructs are themselves wide-reaching and heterogeneous (for a review of heterogeneity in pedagogical content knowledge, see Depaepe et al., 2013; for a review of heterogeneity within conceptualizations of informal formative assessment and teacher noticing, see König et al., 2022). Thus, in line with our goals for this conceptual review, we focus specifically on these constructs' possible connection to inferential accuracy.

### Pedagogical content knowledge

Pedagogical content knowledge (PCK) is a manifest aspect of pedagogy that draws on teacher noticing and is the basis for real-time pedagogical decision-making (Ball, 1993; Ball et al., 2008; Ball and Bass, 2000; Kersting, 2008; König et al., 2022). PCK includes teachers' knowledge of pedagogy, the lesson material, their students, and how students generally learn the material, including the errors and misconceptions indicative of learning processes (Aliustaoglu and Tuna, 2021; Evens et al., 2015).

In line with one comprehensive model of PCK (i.e., the Refined Consensus Model of PCK; for a review, see Chan and Hume, 2019), we conceptualize PCK as existing on three levels and grain-sizes: **collective**, **personal**, and **enacted** PCK (for a review, see Behling et al., 2022). **Collective** PCK encompasses knowledge shared by multiple educators within a subject area and includes knowledge such as general misconceptions in student thinking (e.g., that the human body has only one blood stream; Behling et al., 2022). Research on collective PCK has included surveying content-area experts to map a subject-area (e.g., Vergara et al., 2024) and collective PCK may be cultivated within shared learning spaces such as reading groups (e.g., Cooper et al., 2022).

In contrast, **personal** PCK exists within a single teacher and is the individualized distillation of knowledge from collective PCK through that teacher's specific lens of experience, such as the cultural background of the teacher and of their students. Personal PCK can be articulated by the teacher outside of a pedagogical context, often in interviews or surveys (e.g., Aliustaoglu and Tuna, 2021). In the classroom case study we described earlier, Ball's personal PCK was demonstrated when she recruited a schema relevant to the lesson—specifically, that the disconnect between how the students had learned about fractions (as slices of pizza or pie) and number lines (as consisting only of whole numbers) made the task of labeling a fraction on a number line particularly difficult for students (Ball, 2018). Personal PCK shows a strong association with diagnostic competence (Kramer et al., 2021) and teacher noticing (Dreher and Kuntze, 2015). However, the variability of PCK operationalizations across subject area suggests that this relationship is largely defined within-study and should be considered in context (Alonzo et al., 2019; Vergara et al., 2024).

The third level of PCK, **enacted PCK**, differs from personal PCK in that it is contextualized within a particular pedagogical interaction with specific students: Enacted PCK only exists *during* teaching and is often difficult for teachers to recall and articulate (e.g., “Why did you use X metaphor to answer student Y's question?”—see Sherin et al., 2011). Because these moments occur frequently and are fleeting, they are often viewed as impossible to reason about *post hoc* (Behling et al., 2022). However, one method of peeling open teacher cognition underlying enacted PCK is by examining the relationship between a teacher's accuracy in inferring a student's thoughts during a discretionary space and their proximal enacted PCK, a method similar to the video-recorded lessons often used to study enacted PCK (e.g., Hoth et al., 2018, as well as methods used in inferential accuracy, see Hodges et al., 2015).

Inferential accuracy paradigms may be of particular interest to education researchers as an operationalization of enacted PCK. Although some studies have examined students' post-lesson understanding or quiz scores as correlates of PCK (e.g., Ali et al., 2020), we have not identified any that specifically measured the relationship between a teacher's accuracy in monitoring their students' thoughts during a lesson and their PCK. Within Ball's (2018) case study, the first student's explanation was interrupted by a second student who asked, “why'd you choose seven-eighths?” Ball's decision in this discretionary space to not reprimand the second student for interrupting but instead to allow the first student to respond with her mathematical reasoning reflected Ball's enacted PCK and was based on inferring that the student was demonstrating mathematical curiosity. This constituted an accurate inference made by an expert teacher who was familiar with the class. The variability of teacher responses between novice and expert teachers in moments like these may shed light on how enacted PCK is updated or adjusted during instruction. It may show where inaccuracy is not important (i.e., where the classroom script is sufficient) but also circumstances where inaccuracy may be particularly problematic for enacted PCK.

### Informal formative assessment

We now turn to a second educational construct that intersects with inferential accuracy. Formative assessment encompasses the process by which teachers assess their students' understanding of lesson material (Bennett, 2011). Formative assessment is comprised of both *formal* formative assessment, which includes preplanned, graded demonstrations of student learning such as quizzes, tests, and class projects (Ayala et al., 2008; Black and Wiliam, 1998), and *informal* formative assessment (Ruiz-Primo, 2011), the unplanned and ungraded instances wherein teachers (or other students) encourage students to share their current thinking about the lesson material. We focus on informal formative assessment as an enterprise that draws on inferential accuracy in the classroom.

There are many different conceptualizations of informal formative assessment (IFA) and no strong consensus on a single definition or operationalization (König et al., 2022). Given this heterogeneity, we have chosen to frame our discussion in this section around one commonly-used model of IFA identified by Ruiz-Primo and Furtak (2007)—the “ESRU” cycle. Ruiz-Primo and Furtak describe an “ESRU” cycle thus: the teacher *Elicits* a response from a student on an aspect of the current lesson, the *Student* responds, and the teacher *Recognizes* the response, *Utilizing* the information to inform further cycles of instructional dialogue or to progress through the next portion of the lesson. ESRU cycles are an empirically supported teaching strategy (Leenknecht et al., 2021) for gathering data about a student's level of understanding (a pedagogically-related internal state). These instances often include evaluation from the teacher (e.g., acknowledging that the student correctly understands the material) and/or follow-up to further probe student understanding.

While PCK can be drawn upon to increase teachers' inferential accuracy, IFA within ESRU cycles intersects with inferential accuracy in a different way—as a means of directly eliciting information about students' thoughts. Of course, collecting information about a student's thinking is not the sole purpose of ESRU cycles; asking a student to articulate their thinking is itself a useful pedagogical tool both to reinforce that student's understanding as well as to provide opportunities for students to engage with the ideas of their peers (Tao and Chen, 2024). However, ESRU cycles may bolster instructors' inferential accuracy in two ways: first, by prompting students to directly tell teachers what they are thinking about lesson material; and second, by using ESRU cycles to elicit information that instructors can use to construct and update their inferences about what students are thinking and feeling (and potentially also their general PCK).

In Ball's (2018) case study, she made the first student's thinking explicit at two points during the interaction. First, Ball called on this student to share her initial answer. Ball leveraged her personal PCK to infer why the student made this choice and then Ball again made the student's thinking explicit by asking the student to articulate her reasoning to the class. This sequence differs from most inferential accuracy research, which collects retrospective inferences at either arbitrary intervals or those determined by the target while watching a video-taped social interaction (see Tipsord, 2009, for a rare measure of inferential accuracy driven by the perceiver). In contrast, the timing of Ball's attempts to uncover what the student was thinking used an ESRU cycle as a real-time strategy to facilitate inferential accuracy.

It is important to note that ESRU cycles may not yield entirely accurate information from students about what they are thinking. This may be especially true for non-specific or poorly implemented ESRU cycles (e.g., asking students, “do you get it?”). There are a myriad of social pressures influencing student responses that teachers must consider when assessing the veracity of students' self-reported understanding (for a discussion of how perceptions of possible target dishonesty affect inferential accuracy, see DesJardins and Hodges, 2015). For example, within the context of a math class, a student who experiences math anxiety (Ashcraft, 2002), low belongingness in math (Banchefsky et al., 2019; Good et al., 2012; Lewis and Hodges, 2015), math-related stereotype threat (Spencer et al., 1999), or a combination of the three may seek to conceal a low level of understanding (or perceived low-level of understanding) when responding to a teacher's question, preferring to say, “I don't know” rather than risk the embarrassment of being incorrect. Although classrooms are highly variable, examining teachers' ability to accurately infer students' true state of mind in these contexts may prove a productive path for future researchers.

## Discussion: integrating inferential accuracy into classroom interactions and future questions

Our consideration of inferential accuracy in the context of educational research has likened it to a highly specific form of diagnostic competence and teacher noticing found at the underexamined grain-size level of student thoughts and feelings that may inform teacher choices in discretionary spaces. Prior research has examined teacher cognition during instruction, noting how teachers notice student learning, adapt to challenges, provide scaffolding on the fly, and flexibly support students during instruction (Klug et al., 2016; König et al., 2022; Loibl et al., 2020; Sasse et al., 2025). However, student thoughts and feelings—the unit of analysis examined in inferential accuracy—has received little-to-no attention, with research on teachers' expertise instead focused on post-lesson measures of student learning as the primary outcome measure (Tao and Chen, 2024). However, it is clear in Ball's (2018) case study that how teachers navigate discretionary spaces may also reflect expertise and one method of capturing this skill would be to examine inferential accuracy. Ball's example illustrates something we have aimed to do throughout this paper—that is, to make visible the connections between actions occurring in educational contexts that correspond to predictors of inferential accuracy. In the discussion that follows, we first consider sources of tension when bringing inferential accuracy research into the classroom before next turning to promising future directions where education and social psychology might fruitfully collaborate on inferential accuracy research.

### Tension between inferential accuracy and education

Adapting inferential accuracy research to classroom contexts presents some challenges, although aspects of inferential accuracy paradigms are not entirely novel or foreign in studies of classroom interactions. Videos of classroom teaching are frequently used as stimuli when studying both PCK (Berliner, 2001; Hoth et al., 2018; Kersting, 2008; Kersting et al., 2016; van Es and Sherin, 2021) and IFA (Furtak et al., 2017; Gotwals et al., 2015; Sato et al., 2008; Wells and Arauz, 2006). However, inferential accuracy research differs from the modal study of these other topics in that it collects separate data from the perceiver (teacher) and the target (student). A point of difficulty may be the added vector of where students are developmentally and cognitively (e.g., Tucker-Drob, 2009). High variability in classroom teaching goals and content across grade level suggests that the relationship between inferential accuracy and instruction is likely to be very different across classroom contexts: A university lecturer may speak to a room of 300 students from a lectern 15 feet away from the first row of seats, whereas a teacher in a preschool class may never be more than 15 feet away from any of their 20 students. The importance of individual students' thoughts and feelings, the teacher's individualized knowledge of each student, and pedagogical goals in different classrooms may greatly affect how inferential accuracy relates to the teacher's instruction.

A cautionary note is in order: expecting past inferential accuracy findings based on data collected from dyads in controlled laboratory settings to generalize neatly to classroom settings is probably unreasonable. Researchers may find success in studying inferential accuracy in pedagogical dyads, but the mechanisms and processes underlying inferring the thoughts and feelings for one target may not generalize to the challenge of integrating or generalizing inferences across a whole classroom of students. The degree to which individual discretionary spaces are dyadic and thus amenable to examination is an empirical one. However, when viewing teacher decision-making at the grain-size of discretionary spaces, dyadic interactions may turn out to play an important role.

## Future directions

In this paper, we have tried to lay the groundwork for potential collaboration between social psychology and education around inferential accuracy. Although we believe there are many possible avenues for such collaboration, we now highlight three future directions that may prove particularly fruitful. First, inferential accuracy may aid in distinguishing between the respective contributions of positive teacher-student relationships (e.g., Li et al., 2022) and content area expertise (e.g., Park and Chen, 2012). For example, to what extent is teacher expertise a function of how well a teacher knows their specific students, and does the variance in teacher expertise that is attributable to inferential accuracy help us to know how well teacher expertise may generalize across different subject areas? A math teacher's accuracy at inferring the thoughts and feelings of a familiar student learning material outside the teacher's usual content area (e.g., in a science class) may differ from the inferential accuracy of a science teacher encountering that same student for the first time in a science class. Although prior research on diagnostic competence and teacher noticing would give the advantage to the content-area expert (i.e., the science teacher—see Sherin et al., 2011) due to accuracy at inferring student thoughts germane to the lesson, there may also be a benefit conferred from accuracy at inferring thoughts *irrelevant* to the current lesson material, as this accuracy may contribute to positive teacher-student relationships (Yu et al., 2018).

Second, the study of inferential accuracy in the classroom may also open up new lines of research on how student readability affects instructional and learning success: Some students may be an “open book” to all their teachers, while others' minds may not be easily inferred or may vary in how readable they are based on the teacher or subject area (Lewis, 2014). By comparing student targets across different instructors and subject areas, researchers may find insights into how constructs like test anxiety or stereotype threat may vary in terms of how they impact student readability. For example, is one student highly readable in all their classes except math, where they feel considerable stereotype threat? Is another student an “open book” in class with their favorite instructor, but inscrutable elsewhere? Or perhaps student readability is generally stable across classroom instructor and subject area.

Third, social psychology's study of inferential accuracy research may have a great deal to gain from venturing into instructional settings, including developments in how inferential accuracy is studied and a better understanding of what can improve inferential accuracy. If any group should be proficient at inferring a student's thoughts and feelings during a lesson, it is likely to be teachers. It may be that teachers' PCK and expert schemas for student learning help teachers to make accurate inferences, or it may be that teachers' explicit requests for students to report what they are thinking as part of informal formative assessment provide teachers with better information. These strategies may be adapted to other contexts where people in certain roles need to know what targets are thinking and feeling. There may also be characteristics associated with teachers that help with inferential accuracy, e.g., factors related to self-selection in choosing teaching as a profession and/or a high motivation to accurately infer what students are thinking in order to help students or to succeed at teaching (higher motivation in certain contexts has been previously linked to greater inferential accuracy—see Klein and Hodges, 2001; Thomas and Maio, 2008). Additionally, classroom settings may broaden how inferential accuracy is defined and studied. For example, the skills and knowledge of expert teachers may entail monitoring many students simultaneously, and this ability may offer novel insights that may generalize to other, non-pedagogical contexts in which interactions involve more than a single dyad.

In closing, we have attempted to link research findings from the inferential accuracy literature with teacher practices in educational contexts and to draw connections between predictors of inferential accuracy and several common educational constructs (PCK, IFA). We have pointed out major sources of tension in trying to make these links, such as the difficulty in translating the dyadic methods (a pair of people interacting) that inferential accuracy research has primarily focused on into the classroom, where a teacher is interacting with multiple students. We have described areas of promising future research—some of them directly motivated by the interdisciplinary challenges of merging social psychological approaches in educational contexts. We expect that examining inferential accuracy in educational settings will involve some of the lines of inquiry we have explored here (e.g., PCK, IFA) and likely several others. Classrooms are complex social ecosystems in which teachers engage in specialized social cognition and leverage a diverse skillset to teach effectively. This complexity has resulted in a substantial body of research directed at understanding and improving teacher expertise—research that both may be further informed by incorporating insights from work on inferential accuracy *and* also may generate important new questions and answers about inferential accuracy.

## Footnotes

1 In this paper, we use “dyad” to represent a student-teacher pair with teacher as perceiver and student as target. As we will discuss later, dyadic interactions may occur in full classrooms as long as they predominantly occur between one student and one teacher.

2 The formal social psychological definition of a stereotype is a generalization about members of a group (e.g., Aronson et al., 2010)—these generalizations may or may not be accurate. A *correct* generalization about members of a group (e.g., new mothers are generally tired) would be an accurate stereotype. However, to avoid negative connotations with prejudice and discrimination that are evoked by the use of the word stereotype, we use the term “schema” (which refers to a broad range cognitive constructs that scaffold people's perceptions, including stereotypes) in the current paper.
